# Thermosensitive Chitosan–Gelatin–Glycerol Phosphate Hydrogels as Collagenase Carrier for Tendon–Bone Healing in a Rabbit Model

**DOI:** 10.3390/polym12020436

**Published:** 2020-02-13

**Authors:** Yu-Min Huang, Yi-Cheng Lin, Chih-Yu Chen, Yueh-Ying Hsieh, Chen-Kun Liaw, Shu-Wei Huang, Yang-Hwei Tsuang, Chih-Hwa Chen, Feng-Huei Lin

**Affiliations:** 1 Department of Biomedical Engineering, National Taiwan University, Taipei 100, Taiwan; yellowcorn0326@yahoo.com.hk (Y.-M.H.); judyya1022@gmail.com (S.-W.H.); 2 Department of Orthopedics, Shuang Ho Hospital, Taipei Medical University, Taipei 100, Taiwan; iam4290@gmail.com (Y.-C.L.); aleckc2424@gmail.com (C.-Y.C.); ianhsie@gmail.com (Y.-Y.H.); d92008@yahoo.com.tw (C.-K.L.); tsuangyh@gmail.com (Y.-H.T.); 3 Department of Orthopedics, School of Medicine, College of Medicine, Taipei Medical University, Taipei 100, Taiwan; 4 Department of Orthopedics, Taipei Medical University – Shuang Ho Hospital, School of Medicine, College of Medicine, School of Biomedical Engineering, College of Biomedical Engineering, Research Center of Biomedical Device, Taipei Medical University, Taipei 100, Taiwan; chihhwachen@gmail.com; 5 Institute of Biomedical Engineering & Nanomedicine, National Health Research Institutes, Miaoli County 360, Taiwan

**Keywords:** hydrogel, tendon–bone healing, collagenase digestion, rabbit model

## Abstract

Healing of an anterior cruciate ligament graft in bone tunnel yields weaker fibrous scar tissue, which may prolong an already prolonged healing process within the tendon–bone interface. In this study, gelatin molecules were added to thermosensitive chitosan/**β**-glycerol phosphate disodium salt hydrogels to form chitosan/gelatin/**β**-glycerol phosphate (C/G/GP) hydrogels, which were applied to 0.1 mg/mL collagenase carrier in the tendon–bone junction. New Zealand white rabbit’s long digital extensor tendon was detached and translated into a 2.5-mm diameter tibial plateau tunnel. Thirty-six rabbits underwent bilateral surgery and hydrogel injection treatment with and without collagenase. Histological analyses revealed early healing and more bone formation at the tendon–bone interface after collagenase partial digestion. The area of metachromasia significantly increased in both 4-week and 8-week groups after collagenase treatment (*p* < 0.01). Micro computed tomography showed a significant increase in total bone volume and bone volume/tissue volume in the 8 weeks after collagenase treatment, compared with the control group. Load-to-failure was significantly higher in the treated group at 8 weeks (23.8 ± 8.13 N vs 14.3 ± 3.9 N; *p* = 0.008). Treatment with collagenase digestion resulted in a 66% increase in pull-out strength. In conclusion, injection of C/G/GP hydrogel with collagenase improves tendon-to-bone healing in a rabbit model.

## 1. Introduction

The anterior cruciate ligament (ACL) is one of the most commonly injured soft tissue structures, with an injury incidence of one in 3000 in the American population [1]. Primary repair is not effective for treating injured ACL tissue due to poor healing potential. ACL reconstruction with autografts or allografts has a greater than 90% success rate in restoring knee stability or previous functional performance [2]. Autologous bone-patellar-tendon grafts (BPTB) achieve good results due to direct bone-to-bone healing in femoral and tibial tunnels. However, the harvesting of BPTB results in significant donor morbidity, anterior knee pain, and higher rates of osteoarthritis [3]. Autologous hamstring tendon grafts restore function to a similar extent as BPTB grafts, but have a higher graft failure rate and revision rate [4,5]. Currently, there is an unmet need for interventions that can enhance tendon-to-bone healing to attain a lower graft failure rate and better functional outcomes.

One of the keys to successful ACL reconstruction is healing of the tendon graft to bone. In general, there are transition zones between the tendon-to-bone interface, with four tissue types as follows: tendon, unmineralized fibrocartilage, mineralized fibrocartilage, and bone [6]. Rodeo et al. found a healing process with a fibrovascular interface between the tendon and bone in an extra-articular model [7]. The healing of the tendon-to-bone interface is slow due to the relative avascularity of the fibrocartilage zone and bone loss [8]. The histological arrangement is similar to using Sharpey fibers as indirect insertions, such as in the medial collateral ligament of the knee joint. Because of the scar tissue between the tendon and bone interface, there is a weak connection between tendon grafts and bones in ACL reconstruction, especially in the first six weeks [9]. Besides, tenocytes are surrounded in a dense extracellular matrix (ECM), which limits their mobility and proliferation. Our previous study showed that partial collagenase digestion could improve healing for osteochondral defects in a mice model by degrading the extracellular matrix [10]. Hong et al. also demonstrated the injection of hydrogel, which induced macrophages to produce MMP-9 enzyme for tissue repair by promoting extracellular matrix remodeling [11]. In this present study, we aimed to investigate the effects of low dose collagenase for partial digestion of the ECM in improving tendon–bone healing.

Thermosensitive polymers have gained great attention in drug delivery, cell encapsulation, and tissue engineering [12]. These polymers can be carriers for drug delivery through minimal invasive surgery. Injectable polymers have several advantages in drug delivery, including easy preparation, prolonged and localized drug release, and low systemic toxicity [13]. Chitosan/glycerol phosphate (GP; disodium salt) is one of the widely exploited thermosensitive hydrogels and has drug-delivery potential [14]. Chitosan is an aminopolysaccharide derived from alkaline deacetylation of chitin, which is present in fungal cell walls. In combination with glycerol phosphate, the chitosan/GP hydrogel becomes thermosensitive in diluted acids and can proceed gelation around body temperature [15]. Chitosan/GP hydrogel can be in a liquid state at physiological PH and then a gel when heated at 37 °C. Ahmadi et al. indicated chitosan-GP is a biocompatible hydrogel for mesenchymal stem cell proliferation at certain concentrations [16]. Roughley et al. demonstrated that lack for firm structure of chitosan/GP hydrogel may not be suitable for cell culture [17]. In order to improve the gelation strength, gelatin was added to the thermosensitive chitosan-GP hydrogels. Gelatin is a biocompatible and biodegradable polymer for tissue engineering [18]. Cheng et al. showed chitosan–gelatin–glycerol phosphate hydrogels are biocompatible for nucleus pulposus cell regeneration with better gel strength [19]. When gelatin is added to the chitosan/GP solution at low temperature, hydrogen bonds exist between the OH group of gelatin and the OH and NH_2_ groups of chitosan. Due to high hydrophilicity of gelatin, the binding between gelatin and water can decrease the mobility of chitosan molecules.

The purpose of this study was to determine whether partial collagenase digestion can improve tendon-to-bone healing in a bone tunnel animal model. We hypothesized that the administration of a collagenase hydrogel could degrade the fibrous tissue and lead to the early regeneration of fibrovascular tissue during the healing process. Furthermore, we hypothesized that improved histological results would indicate stronger tendon-to-bone fixation.

## 2. Materials and Methods

### 2.1. Collagenase Hydrogel Preparation

A sterile 0.1% collagenase solution was prepared by mixing 10 mg of Liberase TM Research Grade (Roche, Mannheim, Germany) with 10 mL of Dulbecco’s modified Eagle’s medium (DMEM). An injectable and thermosensitive chitosan/gelatin/glycerol phosphate (C/G/GP) hydrogel was used as a controlled release system for enzyme delivery [19]. For hydrogel preparation, 2.5% chitosan (degree of deacetylation >95%, Kiotek, Taiwan) and 1% gelatin (G1890, Sigma-Aldrich, St. Louis, MO, USA) were dissolved in 0.1 M acetic acid (242,853, Sigma-Aldrich, St. Louis, MO, USA). Glycerol 2-phosphate disodium salt hydrate (β-GP, G6251, Sigma-Aldrich, St. Louis, MO, USA) was dissolved in water and filtered using a 0.22-µm filter (Millex-GV; Millipore, Billerica, MA, USA) for sterilization. The C/G/GP solution was adjusted to pH 7.4, which was suitable for collagenase release. The thermosensitive C/G/GP hydrogel gelled at 37 °C and was injectable with the collagenase preparation.

### 2.2. Cytotoxicity Evaluation

The Achilles tendon was harvested from New Zealand White rabbit following euthanasia. Tendon was washed by PBS buffer (Biochrom, Berlin, Germany) with 100 U/mL penicillin (P4083; Sigma, St. Louis, MO, USA) and cut into small pieces. Minced pieces were placed in a 48-well culture plate and incubated with 0.2% (*v/v*) collagenase (C0130; Sigma, St. Louis, MO, USA) for 30 min. Further DMEM containing 10% fetal bovine serum (Cat. No. 100-106; Gemini Bio-products, West Sacramento, CA, USA) was added and cultured. Tenocytes of passage less than 5 were used in the cytotoxicity test. Tenocytes were seeded in the 96-well cell culture plates (92,096; TPP, Ho Chi Minh City, Vietnam) at a density of 10,000 cells/well and cultured in DMEM mixture F-12 ham medium (DMEM-F12, D8900; Sigma). After incubation for 18 h, the cells were washed with PBS buffer and the solution of hydrogel was added into the culture well (200 μL/well). WST-1 (Cell Proliferation Reagent WST-1; Roche, Mannheim, Germany) was measured at day 1 and 3 to check the cell viability. OD values was measured at 450 nm by using enzyme-linked immune-sorbent assay (ELISA) reader (Sunrise Remote; Tecan, Durham, NC, USA). LDH (CytoTox96 Non-Radioactive Cytotoxicity Assay; Promega, Madison, WI, USA) assay was applied to identify the collagenase C/G/GP hydrogel toxicity to the tenocyte. LDH was measured with a 30 min coupled enzymatic assay and measured using ELISA reader at a wavelength of 490 nm.

### 2.3. Release Profile of Collagenase from C/G/GP Hydrogel

In order to obtain the release profile of collagenase, 1 cc C/G/GP hydrogel was prepared with 0.1 mg/mL collagenase solution. Once the hydrogel gelled completely, the material was immersed in a 24-well plate with 1.5 mL PBS buffer solution/well at 37 °C. The hydrogel soaked in the PBS solution was collected at regular time intervals (30 min, and 1, 3, 8, 24, 48, and 96 h) and refilled with fresh PBS. The PBS obtained was measured by UV-vis spectrometer analysis at 258 nm to quantify the release profile of collagenase from the C/G/GP hydrogel.

### 2.4. Animal Study Design

**Figure 1:**
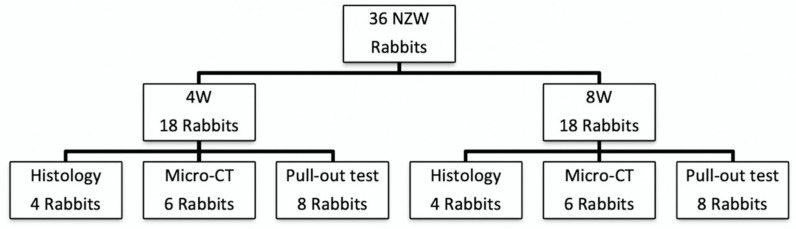
Animal study design.

Thirty-six male New Zealand white rabbits (weighing 3.25 ± 0.15 kg) (from BioLASCO Taiwan Co., Taipei, Taiwan) were used in this study. All the rabbits were randomly allocated to subgroup by a random number generator. The collagenase-containing hydrogel was injected into the right knee and the left knee was injected with C/G/GP hydrogel only without collagenase. Eighteen rabbits were prepared for histological and image evaluation at 4 and 8 weeks, respectively (Figure 1). Four rabbits per group were prepared for histological study, and six rabbits per group were evaluated by microcomputed tomography (micro-CT) analysis. Eight rabbits from each group were subjected to a biomechanical pull-out test.

Flowchart shows the distribution in our animal study.

### 2.5. Surgical Procedure

All procedures were carried out according to the Guide for the Care and Use of Laboratory Animals and approved by Taipei Medical University (LAC-101-0210) on 7^th^ March 2013. All rabbits received a preoperative dose of intramuscular cefazolin sodium (0.1 mg/kg) as a prophylactic antibiotic. Ketamine (40 mg/kg) with xylazine (5 mg/kg) (Rompun; Bayer Healthcare, Leverkusen, Germany) was intramuscularly injected to induce general anesthesia. Using an animal model similar to that developed by Chang et al., [20] the long digital extensor tendon was dissected from the lateral femoral condyle. We created a 2.5-mm-diameter tunnel by electrical drill at the proximal tibial metaphysis at a 30-degree angle from lateral to medial relative to the long bone axis. In our study, the bony tunnel was extra-articular without connection to the knee joint. The long digital extensor tendon was detached from the origin site and translated into the drill hole and fixed with a 3-0 vicryl suture at the medial capsule of the knee joint. Then, 0.5 mL C/G/GP hydrogel with 0.1 mg/mL Liberase TM Research Grade was injected into the bone tunnel through a 24-gauge needle after tendon passed through on right knee. The left knee underwent the same procedure with hydrogel injection without collagenase. The rabbits were allowed to freely exercise in the same cage without restrictions. Euthanasia has been performed under CO2 chamber at 4 and 8 weeks, and the animals were randomly prepared for histological and biomechanical examination.

### 2.6. Histological Examination

The specimens were fixed with 10% formalin for 24 h and decalcified in a graded series of alcohol. The proximal tibia was embedded in paraffin and sliced into 5-µm-thick sections sagittal to the bone tunnel. The samples were sectioned parallel to the longitudinal axis of the tibial bone tunnel. Staining was performed using hematoxylin and eosin (H&E) for the identification of collagenous fiber tissue. Histological sections were examined using a light microscope. Healing between the tendon and the bone tunnel was assessed by identifying new tissue (woven bone formation or fibrovascular granulation tissue).

The area of fibrovascular tissue at the bone-tendon interface was identified as the area of metachromasia. The total area of metachromasia was measured for all histological specimens using Image J imaging software. The area of metachromasia was gauged by two independent observers for histological measurements.

### 2.7. Micro-Computed Tomography Evaluation

At 4 and 8 weeks after surgery, six rabbits from each group were sacrificed. The specimens were prepared for micro-CT analysis (Skyscan 1176 µCT System; Bruker, Kontich, Belgium). A 9-µm resolution along the long axis of the tibial bone tunnel was acquired using a consecutive micro-CT imager (50 kV X-ray voltage and 200 µA electric current). The scans yielded reconstructed 3D data sets with a voxel size of 9 µm, which were evaluated using a CT automated image analysis system (Bruker, Kontich, Belgium). The volume of interest (VOI) contained the whole bone tunnel with the tendon graft and the surroundings. Total bone volume (TBV, mm^3^) and the bone volume fraction (BV/TV) were measured from the number of bone voxels and the total voxel number in the VOI. Trabecular thickness (TbTh, µm) was measured from the trabecular architecture around the tendon graft. Bone mineral density (BMD, g/mm^3^) was also calculated using the analysis system. The two investigators who evaluated the CT data were blinded to the animal study.

### 2.8. Biomechanical Testing

Eight rabbits from each group were selected for biomechanical testing.

Before testing, we carefully dissected the tendon from the distal end of the insertion and removed all the other soft tissue. We used a Bose ElectroForce 3510 Fatigue Tester at an elongation rate of 10 mm/min. The graft was secured on the clamps 3 cm away from the lateral tibial aperture, allowing tensile loading along the long axis of the tibial bone tunnel. Following a static preload of 1 N for 2 min, ultimate load-to-failure in uniaxial tension at 20 mm per minute was determined from the load-deformation curve. The load-deformation curve was recorded, from which the ultimate load-to-failure and stress (load/cross-section area) of the tendon graft were recorded.

### 2.9. Statistical Analyses

Before the animal study, a power analysis was performed to determine the number of animals needed to prevent a type II error. In a previous study, Gulotta et al. showed that the TBV determined by µCT imaging was 27.0 ± 8.1 mm^3^ for experimental tibia [20]. Five specimens per group provided a power of 0.80 with α = 0.05. For biomechanical testing in this study, we performed a pilot study at our institution that showed that the ultimate pull-out strength was 13.1 ± 8.4 N". This analysis showed that 16 specimens (8 specimens in each group) could achieve a power of 0.80 with α = 0.05. The obtained biomechanical data and CT analysis were compared using paired t-tests. All statistical analyses were performed using SPSS 18.0 software (SPSS Inc., Chicago, IL, USA). *p* < 0.05 was considered statistically significant.

## 3. Results

### 3.1. Cytotoxicity

**Figure 2:**
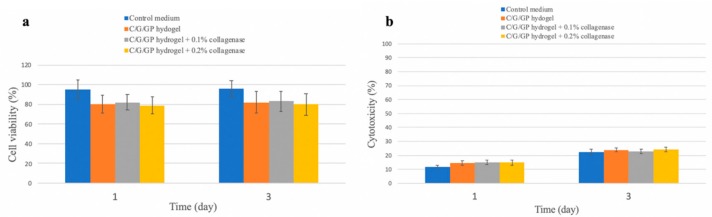
Cytotoxic of chitosan/gelatin/**β**-glycerol phosphate (C/G/GP) with collagenase to tenocyte. Figure 2a shows water soluble tetrazolium salt-1 (WST-1) assay (n = 5, *p >* 0.05) under different collagenase concentration and Figure 2b shows lactate dehydrogenase assay (n = 5, *p* > 0.05).

Figure 2a shows the results of WST-1 assay of the C/G/GP hydrogel with collagenase at day 1 and 3. There is no significant difference between the C/G/GP hydrogels with or without collagenase, compared with the monolayer cultured group (n = 5, *p*> 0.05). Figure 2b shows the LDH assay among control medium and C/G/GP hydrogels groups. The results of LDH assay show no significant difference among experimental agents (n = 5, *p* > 0.05). The results of LDH assay illustrate the C/G/GP hydrogel with collagenase has no cytotoxic effect on tenocyte. Hence, we choose the C/G/GP hydrogel with 0.1% collagenase for our experiments according to the safety test.

### 3.2. Collagenase Release Profile

**Figure 3:**
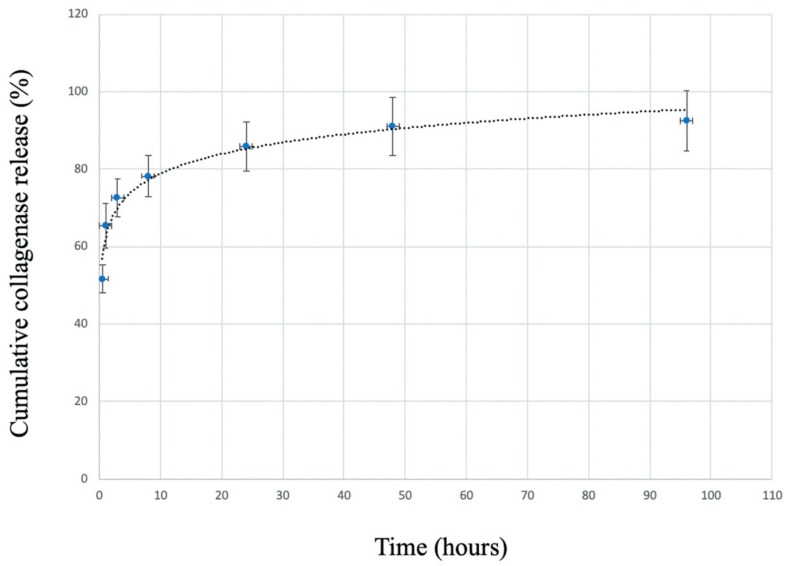
Release profile of the collagenases loaded in C/G/GP hydrogel. The release profile was evaluated by incubation C/G/GP hydrogel in PBS with collagenase concentration of 0.1 mg/mL. The error bars designed the standard deviation.

The collagenase release profile from the C/G/GP hydrogel is given in Figure 3. The release profile was observed at the quick release in the first 30 min, and at sustained release until 4 days. Fifty-one percent collagenase was released from hydrogel in the first 30 min, and saturation was continued after 48 h. The profile illustrates that collagenase could release faster and reach higher cumulative release rate (92.5%).

### 3.3. Gross Observations

**Figure 4:**
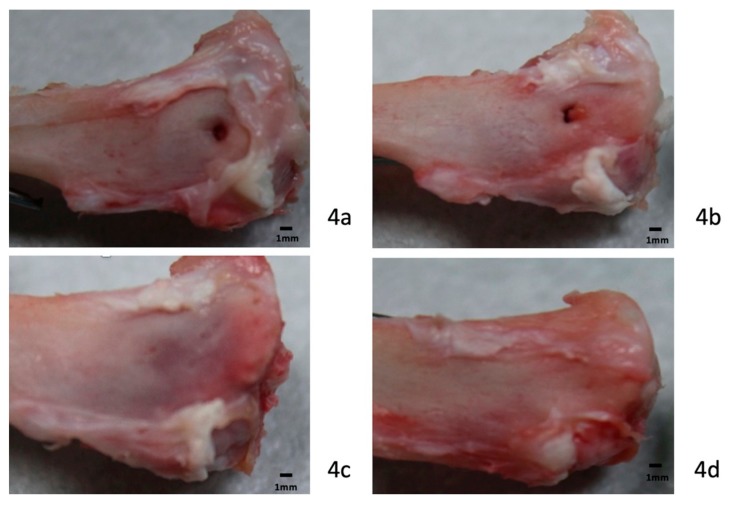
Necropsy examination. Photography at necropsy showed bone-tunnel healing in a rabbit model. A bony defect was identified in the control group at 4 weeks (**a**) and 8 weeks (**b**). Better bony ingrowth with a decrease in tunnel diameter was seen in the study group at 4 weeks (**c**) and 8 weeks (**d**) (scale bar: 1 mm).

After necropsy, all specimens were examined from the knee joint to the middle aspect of the tibia. The knee joint contained clear synovial fluid without signs of infection. Synovitis with hypertrophy of the synovium was not observed in this study. Gross observation revealed no evidence of an adverse effect after using collagenase digestion in the knee joint or surroundings. Soft tissue around the bone tunnel at the tibial metaphysis showed no obvious necrosis or defects in either group. The bony defect in the bone tunnel was clearly identified in the control group (Figure 4a,b). In the control group, only one specimen was found to have bone formation in the bone orifice at 4 weeks, and six specimens were observed to have bone formation at 8 weeks. Bone tunnel healing with new bone formation was observed in all study groups, especially in the 8-week groups (Figure 4c,d). All surgical wounds healed smoothly, without wound dehiscence or infection.

### 3.4. Histology

#### 3.4.1. Four-Week Specimens

**Figure 5:**
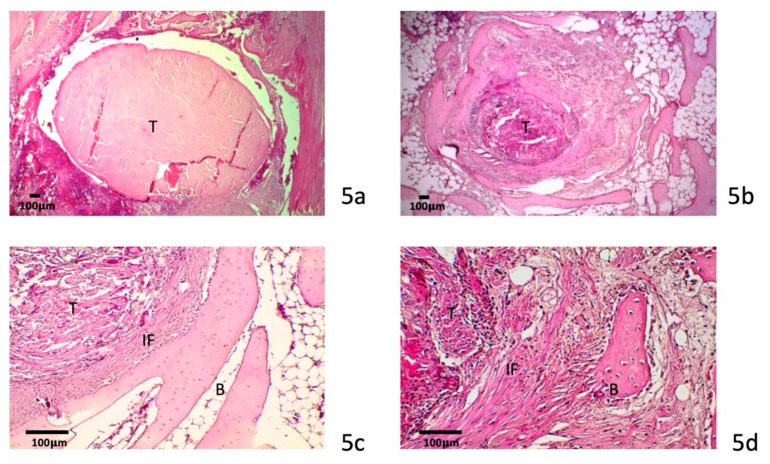
Four-week specimens histology. Histology of a tendon (T)-to-bone (B) interface (IF) in a 4-week specimen in the control group (**a**) and the study group (**b**). A wider interface with dense fibrovascular tissue formation in the study group was found. Sharpey fiber-like tissue was more observed 4 weeks after collagenase treatment (**c**,**d**) (scale bar: 100 μm).

Healing at the tendon–bone interface was observed with the formation of fibrovascular tissue in the treated group (Figure 5a,b). A large gap between the tendon and bone was clearly identified in the control group. More dense fibrous tissue formation was observed at both ends of the bone tunnel, especially in the medial orifice. There was very little bone formation and cartilage observed around the bone tunnel interface in the control group specimens.

In the collagenase digestion group, more maturation of the fibrous tissue was observed at the tendon–bone interface (Figure 5c,d). The morphology and structure of the tendon graft showed no significant difference from the control group. There was no evidence of a foreign body giant cell response in the treated group. Fibrovascular tissue at the tendon–bone interface was more mature in the collagenase-treated group than in the control group. Sharpey fiber-like tissue was also identified at tendon–bone interface. Incorporation of the tendon with collagen fibers and bone was more prominent in the treated specimens than in the control specimens. Proliferation of fibrous tissue at the tendon–bone interface was more advanced and apparent in the treated group than in the control group.

#### 3.4.2. Eight-Week Specimens

**Figure 6:**
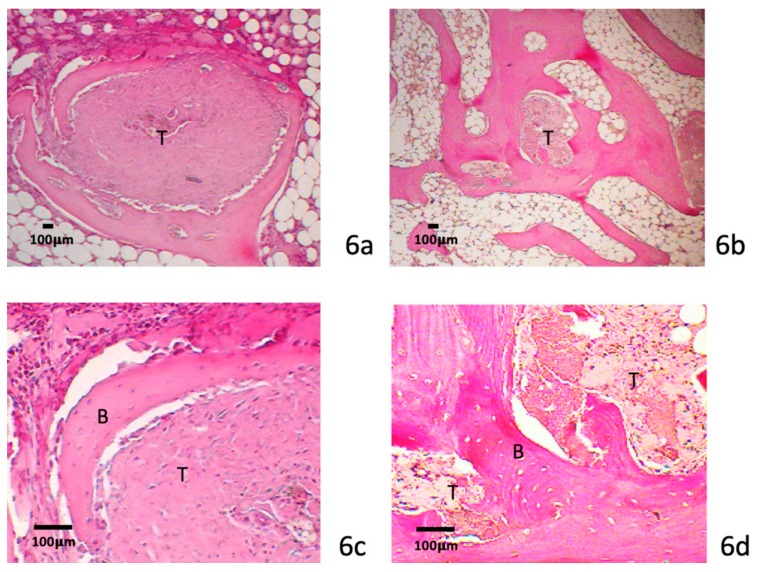
Eight-week specimens histology. Wider dense interface tissue between the tendon (T) and bone (B) in the 8-week study group (**a**,**b**). New bone formation with incorporation into the tendon graft is illustrated (**c**,**d**). The tendon graft was replaced by new bone formation after collagenase treatment. (scale bar: 100 μm).

At 8 weeks, there was more mature fibrovascular tissue incorporated at the tendon–bone interface after collagenase treatment. More Sharpey fiber-like tissue was observed in the treated specimens than the 4-week specimens. More bone formation with incorporation into the tendon graft was observed in the treated group than in the control group (Figure 6a,b). The tendon graft volume was decreased with early woven bone integration (Figure 6c,d). All treated specimens showed aggressive bone ingrowth at both ends of the bone tunnels. There was no significant difference in bone ingrowth between the medial and lateral orifice. The treated specimens had a wide interface zone and more woven bone formation than the control group.

#### 3.4.3. Metachromasia

**Figure 7:**
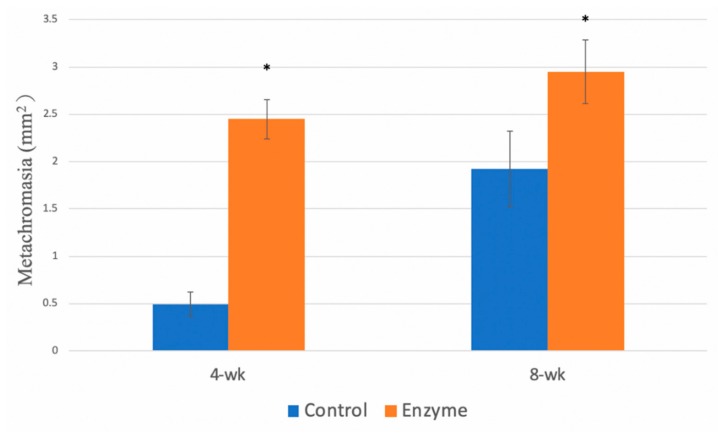
Metachromacia. The area of metachromcia was significantly increased in both 4-week and 8-week groups after collagenase treatment (*p* < 0.01). The error bars designed the standard deviation.

Histomorphometric analysis showed a significantly greater area of fibrovascular tissue at the tendon–bone interface in the collagenase group (Figure 7). Early and significant fibrous healing was demonstrated in the 4-week group (*p* = 0.0008). Significant increase of metachromacia was also demonstrated in 8-week group after collagenase treatment comparing with control group (*p* = 0.004).

### 3.5. Micro-Computed Tomography Evaluation

**Figure 8:**
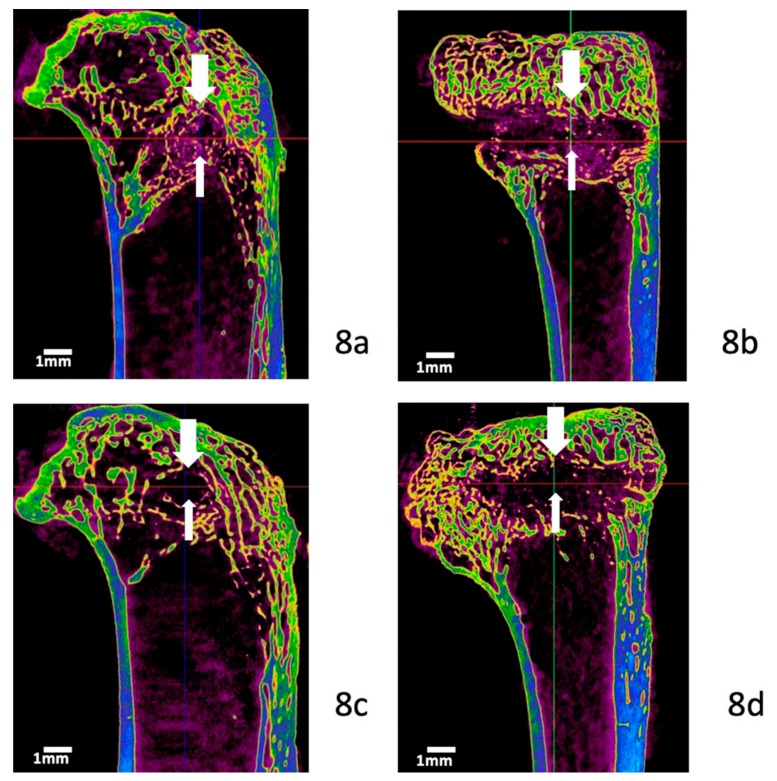
Microcomputed tomography (Micro-CT) examination showed bone formation. Micro-CT shows tendon graft and bone tunnel in an 8-week specimen from the control group. (**a**,**b**) Bone tunnel obliteration with new bone ingrowth was identified in sagittal and coronal views after enzyme treatment (**c**,**d**). Prominent bone ingrowth was observed in both tunnel orifices. Line arrow marks the tendon graft, and block arrows indicate the bone tunnel. (scale bar: 1 mm).

**Figure 9:**
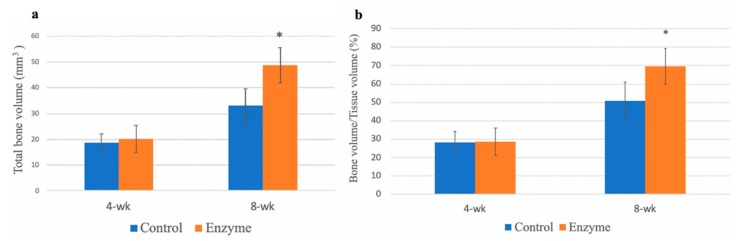
Micro-CT evaluation. Total bone volume (mm^3^ ± SD) determined by micro-CT. The region of interest was measured following the long axis of the bone tunnel. (**a**) There was significantly more new bone formation at 8 weeks after collagenase treatment. Total bone volume/tissue volume was illustrated, and the ratio was significant 8 weeks after enzyme treatment. (**b**) *p* < 0.05, n = 6. The error bars designed the standard deviation.

New bone formation was evaluated by micro-CT, and the TBV was quantified. 3D reconstruction images showed more new bone formation in the treated specimens than in the control specimens at 8 weeks. In the control group, the bone tunnel with tendon graft was clearly identified in the CT images (Figure 8a,b). New bone formation with bone tunnel elimination was identified after collagenase treatment (Figure 8c,d). The bony tunnel area was decreased in the sagittal view after collagenase treatment. The new bone formation was prominent near the medial aperture in the coronal view. At 8 weeks, the TBV was significantly greater (*p* = 0.003) in the collagenase group (48.7 ± 6.8 mm^3^) than in the control group (33 ± 6.6 mm^3^) (Figure 9a). However, there were no differences in the TBV between the two groups at the 4-week timepoint. The percentage of new bone formation in the tendon–bone junction was analyzed as bone volume/tissue volume (BV/TV). We also noted the BV/TV was significantly greater (*p* = 0.01) in the collagenase group (69.5 ± 9.8%), compared with control group (50.8 ± 10.1%) at 8-week group. (Figure 9b). In addition, there were no significant differences in the trabecular thickness and BMD between the two groups.

### 3.6. Biomechanical Testing

**Figure 10:**
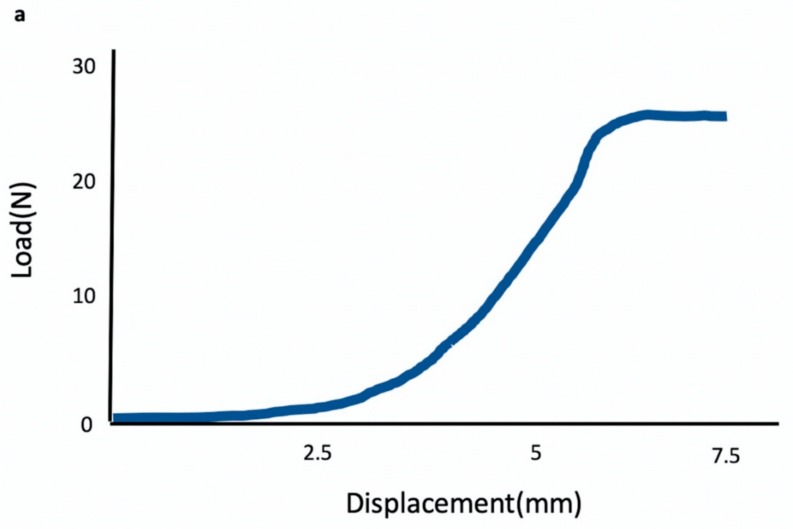

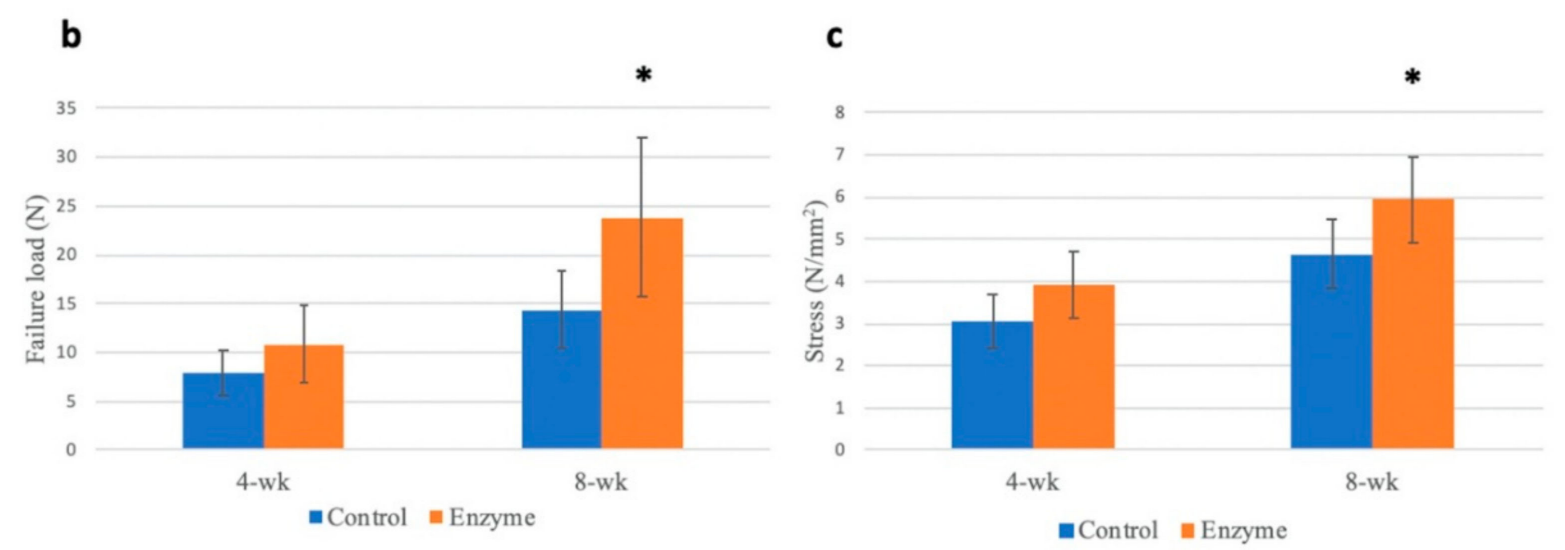
Biomechanical assessment. Original load-deformation curve in the 8-week enzyme-treated specimen. (**a**) Significantly higher tendon ultimate load-to-failure and stress were found in the enzyme treatment group than in the control group at 8 weeks. * *p* < 0.05, n = 8. (**b**,**c**) The error bars designed the standard deviation.

Figure 10a demonstrated the original load-deformation curve in the 8-week enzyme-treated specimen. The ultimate load-to-failure and stress in the treated specimens were greater than that in the control group at 8 weeks. The mean maximal load at failure was 14.3 ± 3.9 N for the control group and 23.8 ± 8.13 N for the collagenase-treated group (*p* = 0.008). The stress was 4.65 ± 0.8 N/mm^2^ for the control group comparing with 5.93 ± 1.02 N/mm^2^ after collagenase treatment. (*p* = 0.03) Treatment with partial collagenase digestion resulted in an increased load of 66%. Most specimens failed in the bony tunnel, and only two specimens (2/8) failed near the proximal orifice in the collagenase group at 8 weeks. There were no differences between the two groups at 4 weeks.

## 4. Discussion

Clinical outcomes after ACL reconstruction using soft tissue grafts are heavily dependent on secure integration at the tendon–bone interface. However, healing of a tendon graft in the bone tunnel involves fibrous scar tissue, which is mechanically inferior to a normal tendon insertion [21,22]. Bedi et al. reported differences in the graft healing process between the intra-articular and extra-articular ends of a bone tunnel [23]. Smith et al. showed more mature tendon-to-bone healing process can occur through a similar four-zone of integration in soft tissue grafts in the canine model [24]. The interface between the tendon and the bone tunnel is the weakest area for six weeks immediately after ACL reconstruction [9]. For these reasons, there is substantial interest in evaluating the effects of different agents on tendon–bone healing. Several experimental agents have been investigated, including recombinant human bone morphogenetic protein-2 (BMP-2) [7], transforming growth factor-ß1 (TGF-ß1) [25], magnesium-based bone adhesive [20], demineralized bone matrix [26], and calcium phosphate-hybridized tendon grafts [27]. Rodeo et al. demonstrated BMP-2 application can enhance bone ingrowth at the tendon–bone interface in a dog model [7]. Yamazaki et al. showed exogenous administration of TGF-**β**1 increased the bonding strength of the graft to the tunnel [25], and Mutsuzaki et al. developed a goat animal model using calcium phosphate hybridization with tendon graft to improve tendon–bone healing [27]. Yoon et al. found local PTH administration with an absorbable scaffold improved the biomechanical and histological outcomes in a rat model [28].

In this study, we evaluated the effect of partial collagenase digestion on tendon–bone interface healing. In the histological examination, we found more new bone formation and early perpendicular collagen fiber integration in the treated group. The appearance of new bone formation indicated a strengthening of the attachment at the tendon–bone interface, and this was confirmed by the mechanical pull-out test. By 8 weeks, the ultimate load-to-failure increased by approximately 66% in the treated group compared to that in the control group. We also used micro-CT to objectively determine the amount of new bone formation. New bone formation found at both ends of the bone tunnel indicated the effect of partial collagenase digestion.

The collagenase used in this study was Liberase TM Research Grade, which contains purified collagenase isoforms I and II and a neutral protease. Native collagenases used for tissue remodeling in humans are matrix metalloproteinases (MMPs). MMPs are a family of zinc-dependent endopeptidases that degrade the extracellular matrix between cells and adjacent tissues [29]. MMPs and their endogenous inhibitors play important roles in maintaining the integrity of the extracellular matrix [30]. The loss of balance between MMPs and their endogenous inhibitors results in degenerative tendinopathy or tendon rupture [31,32,33,34,35]. Bedi et al. reported that local delivery of MMP inhibitors improves tendon–bone healing after rotator cuff repair in a rabbit animal model [36]. In ACL rupture cases, the MMP-1 gene expression level is the highest in the synovium, and the highest level of MMP-13 gene expression is found in the ACL [37]. Demirag et al. reported the enhancement of tendon-to-bone healing by inhibiting MMPs in an ACL reconstruction rabbit model [38]. This suggests that both anabolic and catabolic factors are important in the healing process after ACL reconstruction.

There are two possible mechanisms by which partial collagenase digestion benefits healing. First, the tendon–bone interface is mainly filled with fibrous scar tissue. This fibrous scar tissue is weak, and there is poorer biomechanical resistance than in normal tendon–bone healing. Therefore, collagenase injection into the bone tunnel may digest the fibrous scar tissue and improve native woven bone formation. This was shown in the histology examination of the treated group in both 4-weeks and 8-weeks group. The second mechanism that may improve healing is woven bone overgrowth. In our study, early woven bone was observed in the CT images and histology examination of the treated group. Paiva et al. reviewed the pivotal functions of MMPs during development and bone regeneration in a knockout mouse model [39]. Bone remodeling is mediated by osteoclast recruitment, differentiation, and bone matrix resorption, followed by the recruitment and deposition of osteoblasts for new bone formation [40]. We hypothesized that the injection of a collagenase hydrogel alters the homeostasis of bone remodeling for early woven bone formation.

We acknowledge several limitations in our study. First, we used Liberase TM Research Grade as our experimental agent. Collagenase has been used to induce tendon degeneration or to release tenocytes in a previous study [41]. We used a C/G/GP hydrogel as a collagenase release system. The concentration of collagenase was evaluated in our laboratory, but the ideal amount of collagenase remains uncertain. Second, we conducted a power analysis to determine the numbers of animals required to minimize the concern of a type II error. Significant differences were identified in the 8-week groups, but CT parameters and biomechanical results showed no significant differences at 4 weeks. Therefore, type II errors could have biased this study. Finally, this was a preliminary study to evaluate the effect of partial collagenase digestion on tendon–bone healing. This study was an extra-articular graft model, which was different from ACL reconstruction. A positive effect could not be confirmed for ACL reconstruction surgery. Therefore, further animal studies using this model for ACL reconstruction are warranted.

## 5. Conclusions

Chitosan/gelatin/**β**-glycerol phosphate (C/G/GP) hydrogels was proposed as an ideal carrier for collagenase application to improve tendon–bone healing in a rabbit animal. To achieve a rapid tendon–bone healing, collagenase was incorporated with C/G/GP hydrogel could induce early and more woven bone formation. Biomechanical test revealed higher ultimate load-to-failure and stress after collagenase treatment. Histological examination and micro-CT observed more bone formation and perpendicular collagen fibers after the enzyme treatment. Our findings suggest a simple method and could be used to augment tendon-to-bone healing and call for further research.

## References

[1] Frank C.B., Jackson D.W. (1997). Current concepts review—The science of reconstruction of the anterior cruciate ligament. J. Bone Jt. Surg..

[2] Freedman K.B., D’Amato M.J., Nedeff D.D., Kaz A., Bach B.R. (2003). Arthroscopic anterior cruciate ligament reconstruction: A metaanalysis comparing patellar tendon and hamstring tendon autografts. Am. J. Sports Med..

[3] Poehling-Monaghan K.L., Salem H., Ross K.E., Secrist E., Ciccotti M.C., Tjoumakaris F., Ciccotti M.G., Freedman K.B. (2017). Long-term outcomes in anterior cruciate ligament reconstruction: A systematic review of patellar tendon versus hamstring autografts. Orthop. J. Sports Med..

[4] Gifstad T., Foss O.A., Engebretsen L., Lind M., Forssblad M., Albrektsen G., Drogset J.O. (2014). Lower risk of revision with patellar tendon autografts compared with hamstring autografts: A registry study based on 45,998 primary ACL reconstructions in Scandinavia. Am. J. Sports Med..

[5] Samuelsen B.T., Webster K.E., Johnson N.R., Hewett T.E., Krych A.J. (2017). Hamstring Autograft versus Patellar Tendon Autograft for ACL Reconstruction: Is There a Difference in Graft Failure Rate? A Meta-analysis of 47,613 Patients. Clin. Orthop. Relat. Res..

[6] Cooper R.R., Misol S. (1970). Tendon and ligament insertion. A light and electron microscopic study. J. Bone Jt. Surg..

[7] Rodeo S.A., Suzuki K., Deng X.-H., Wozney J., Warren R.F. (1999). Use of Recombinant Human Bone Morphogenetic Protein-2 to Enhance Tendon Healing in a Bone Tunnel. Am. J. Sports Med..

[8] Wong M.W.N., Qin L., Tai J.K.O., Lee S.K.M., Leung K.S., Chan K.M. (2004). Engineered allogeneic chondrocyte pellet for reconstruction of fibrocartilage zone at bone-tendon junction? A preliminary histological observation. J. Biomed. Mater. Res..

[9] Tomita F., Yasuda K., Mikami S., Sakai T., Yamazaki S., Tohyama H. (2001). Comparisons of intraosseous graft healing between the doubled flexor tendon graft and the bone-patellar tendon-bone graft in anterior cruciate ligament reconstruction. Arthroscopy.

[10] Liao C.-J., Lin Y.-J., Chiang H., Chiang S.-F., Wang Y.-H., Jiang C.-C. (2007). Injecting partially digested cartilage fragments into a biphasic scaffold to generate osteochondral composites in a nude mice model. J. Biomed. Mater. Res. A.

[11] Hong L.T.A., Kim Y.-M., Park H.H., Hwang D.H., Cui Y., Lee E.M., Yahn S., Lee J.K., Song S.-C., Kim B.G. (2017). An injectable hydrogel enhances tissue repair after spinal cord injury by promoting extracellular matrix remodeling. Nat. Commun..

[12] Chen G., Hoffman A.S. (1995). Graft copolymers that exhibit temperature-induced phase transitions over a wide range of pH. Nature.

[13] Lin Z., Gao W., Hu H., Ma K., He B., Dai W., Wang X., Wang J., Zhang X., Zhang Q. (2014). Novel thermo-sensitive hydrogel system with paclitaxel nanocrystals: High drug-loading, sustained drug release and extended local retention guaranteeing better efficacy and lower toxicity. J. Control. Release.

[14] Ruel-Gariépy E., Chenite A., Chaput C., Guirguis S., Leroux J. (2000). Characterization of thermosensitive chitosan gels for the sustained delivery of drugs. Int. J. Pharm..

[15] Chenite A., Chaput C., Wang D., Combes C., Buschmann M.D., Hoemann C.D., Leroux J.C., Atkinson B.L., Binette F., Selmani A. (2000). Novel injectable neutral solutions of chitosan form biodegradable gels in situ. Biomaterials.

[16] Ahmadi R., de Bruijn J.D. (2008). Biocompatibility and gelation of chitosan-glycerol phosphate hydrogels. J. Biomed. Mater. Res. A.

[17] Roughley P., Hoemann C., DesRosiers E., Mwale F., Antoniou J., Alini M. (2006). The potential of chitosan-based gels containing intervertebral disc cells for nucleus pulposus supplementation. Biomaterials.

[18] Huang Y., Onyeri S., Siewe M., Moshfeghian A., Madihally S.V. (2005). In vitro characterization of chitosan-gelatin scaffolds for tissue engineering. Biomaterials.

[19] Cheng Y.-H., Yang S.-H., Su W.-Y., Chen Y.-C., Yang K.-C., Cheng W.T.-K., Wu S.-C., Lin F.-H. (2010). Thermosensitive chitosan-gelatin-glycerol phosphate hydrogels as a cell carrier for nucleus pulposus regeneration: An in vitro study. Tissue Eng. Part A.

[20] Gulotta L.V., Kovacevic D., Ying L., Ehteshami J.R., Montgomery S., Rodeo S.A. (2008). Augmentation of tendon-to-bone healing with a magnesium-based bone adhesive. Am. J. Sports Med..

[21] Goradia V.K., Rochat M.C., Kida M., Grana W.A. (2000). Natural history of a hamstring tendon autograft used for anterior cruciate ligament reconstruction in a sheep model. Am. J. Sports Med..

[22] Grana W.A., Egle D.M., Mahnken R., Goodhart C.W. (1994). An analysis of autograft fixation after anterior cruciate ligament reconstruction in a rabbit model. Am. J. Sports Med..

[23] Bedi A., Kawamura S., Ying L., Rodeo S.A. (2009). Differences in tendon graft healing between the intra-articular and extra-articular ends of a bone tunnel. HSS J..

[24] Smith P.A., Stannard J.P., Pfeiffer F.M., Kuroki K., Bozynski C.C., Cook J.L. (2016). Suspensory Versus Interference Screw Fixation for Arthroscopic Anterior Cruciate Ligament Reconstruction in a Translational Large-Animal Model. Arthroscopy.

[25] Yamazaki S., Yasuda K., Tomita F., Tohyama H., Minami A. (2005). The effect of transforming growth factor-β1 on intraosseous healing of flexor tendon autograft replacement of anterior cruciate ligament in dogs. Arthrosc. J. Arthrosc. Relat. Surg..

[26] Lovric V., Chen D., Yu Y., Oliver R.A., Genin F., Walsh W.R. (2012). Effects of Demineralized Bone Matrix on Tendon-Bone Healing in an Intra-articular Rodent Model. Am. J. Sports Med..

[27] Mutsuzaki H., Fujie H., Nakajima H., Fukagawa M., Nomura S., Sakane M. (2016). Effect of Calcium Phosphate–Hybridized Tendon Graft in Anatomic Single-Bundle ACL Reconstruction in Goats. Orthop. J. Sports Med..

[28] Yoon J.P., Chung S.W., Jung J.W., Lee Y.S., Kim K.I., Park G.Y., Kim H.M., Choi J.H. (2019). Is a Local Administration of Parathyroid Hormone Effective to Tendon-to-Bone Healing in a Rat Rotator Cuff Repair Model?. J. Orthop. Res..

[29] Talhouk R.S., Bissell M.J., Werb Z. (1992). Coordinated expression of extracellular matrix-degrading proteinases and their inhibitors regulates mammary epithelial function during involution. J. Cell Biol..

[30] Bramono D.S., Richmond J.C., Weitzel P.P., Kaplan D.L., Altman G.H. (2004). Matrix metalloproteinases and their clinical applications in orthopaedics. Clin. Orthop. Relat. Res..

[31] De Mos M., van El B., DeGroot J., Jahr H., van Schie H.T.M., van Arkel E.R., Tol H., Heijboer R., van Osch G.J.V.M., Verhaar J.A.N. (2007). Achilles tendinosis: Changes in biochemical composition and collagen turnover rate. Am. J. Sports Med..

[32] Jones G.C., Corps A.N., Pennington C.J., Clark I.M., Edwards D.R., Bradley M.M., Hazleman B.L., Riley G.P. (2006). Expression profiling of metalloproteinases and tissue inhibitors of metalloproteinases in normal and degenerate human achilles tendon. Arthritis Rheum..

[33] Lavagnino M., Arnoczky S.P., Egerbacher M., Gardner K.L., Burns M.E. (2006). Isolated fibrillar damage in tendons stimulates local collagenase mRNA expression and protein synthesis. J. Biomech..

[34] September A.V., Cook J., Handley C.J., van der Merwe L., Schwellnus M.P., Collins M. (2009). Variants within the COL5A1 gene are associated with Achilles tendinopathy in two populations. Br. J. Sports Med..

[35] Treviño E.A., McFaline-Figueroa J., Guldberg R.E., Platt M.O., Temenoff J.S. (2018). Full-thickness rotator cuff tear in rat results in distinct temporal expression of multiple proteases in tendon, muscle, and cartilage. J. Orthop. Res..

[36] Bedi A., Kovacevic D., Hettrich C., Gulotta L.V., Ehteshami J.R., Warren R.F., Rodeo S.A. (2010). The effect of matrix metalloproteinase inhibition on tendon-to-bone healing in a rotator cuff repair model. J. Shoulder Elb. Surg..

[37] Haslauer C.M., Proffen B.L., Johnson V.M., Murray M.M. (2014). Expression of modulators of extracellular matrix structure after anterior cruciate ligament injury. Wound Repair Regen.

[38] Demirag B., Sarisozen B., Ozer O., Kaplan T., Ozturk C. (2005). Enhancement of tendon-bone healing of anterior cruciate ligament grafts by blockage of matrix metalloproteinases. J. Bone Jt. Surg..

[39] Paiva K.B.S., Granjeiro J.M. (2014). Bone tissue remodeling and development: Focus on matrix metalloproteinase functions. Arch. Biochem. Biophys..

[40] Kylmaoja E., Nakamura M., Tuukkanen J. (2016). Osteoclasts and Remodeling Based Bone Formation. Curr. Stem. Cell Res. Ther..

[41] Chen Y.-J., Wang C.-J., Yang K.D., Kuo Y.-R., Huang H.-C., Huang Y.-T., Sun Y.-C., Wang F.-S. (2004). Extracorporeal shock waves promote healing of collagenase-induced Achilles tendinitis and increase TGF-beta1 and IGF-I expression. J. Orthop. Res..

